# Injectable hyaluronic acid-based antibacterial hydrogel adorned with biogenically synthesized AgNPs-decorated multi-walled carbon nanotubes

**DOI:** 10.1007/s40204-021-00155-6

**Published:** 2021-03-26

**Authors:** Pooyan Makvandi, Milad Ashrafizadeh, Matineh Ghomi, Masoud Najafi, Hamid Heydari Sheikh Hossein, Ali Zarrabi, Virgilio Mattoli, Rajender S. Varma

**Affiliations:** 1 Istituto Italiano di Tecnologia, Centre for Materials Interface, viale Rinaldo Piaggio 34, 56025 Pontedera, Pisa Italy; 2 Department of Basic Science, Faculty of Veterinary Medicine, University of Tabriz, 51666-16471 Tabriz, Iran; 3 Sabanci University Nanotechnology Research and Application Center (SUNUM), 34956 Tuzla, Istanbul, Turkey; 4 Chemistry Department, Faculty of Science, Shahid Chamran University of Ahvaz, 61537-53843 Ahvaz, Iran; 5 Medical Technology Research Center, Institute of Health Technology, Kermanshah University of Medical Sciences, 6715847141 Kermanshah, Iran; 6 Radiology and Nuclear Medicine Department, School of Paramedical Sciences, Kermanshah University of Medical Sciences, Kermanshah, Iran; 7 Department of Biotechnology, Faculty of Biological Science and Technology, University of Isfahan, Isfahan, Iran; 8 Regional Centre of Advanced Technologies and Materials, Palacky University, Slechtitelu 27, 783 71 Olomouc, Czech Republic

**Keywords:** Ag NPs, Green synthesis, *Camellia sinensis*, Antibacterial, Nanomedicine, Injectable nanocomposite, Thermosensitive hydrogels

## Abstract

Injectable materials have shown great potential in tissue engineering applications. However, bacterial infection is one of the main challenges in using these materials in the field of regenerative medicine. In this study, biogenically synthesized silver nanoparticle-decorated multi-walled carbon nanotubes (Ag/MWCNTs) were deployed for adorning biogenic-derived AgNPs which were subsequently used in the preparation of thermosensitive hydrogels based on hyaluronic acid encompassing these green-synthesized NPs. The antibacterial capacity of AgNPs decorated on MWCNTs synthesized through *Camellia sinensis* extract in an organic solvent-free medium displayed a superior activity by inhibiting the growth of Gram-negative (*E. coli* and Klebsiella) and Gram-positive (*S. aureus* and *E. faecalis*). The injectable hydrogel nanocomposites demonstrated good mechanical properties, as well. The thermosensitive hyaluronic acid-based hydrogels also exhibited T_gel_ below the body temperature, indicating the transition from liquid-like behavior to elastic gel-like behavior. Such a promising injectable nanocomposite could be applied as liquid, pomade, or ointment to enter wound cavities or bone defects and subsequently its transition in situ to gel form at human body temperature bodes well for their immense potential application in the biomedical sector.

## Introduction

Much attention has been paid to temperature-sensitive hydrogels in recent years in pharmaceutical industry as these hydrogels can be used in liquid or ointment form, easily enter wound cavities, or repair bone defects, and subsequently become in situ gel at human body temperature (Abbadessa et al. 2016; Yu et al. 2017). Amphiphilic block copolymers such as polyethylene oxide–polypropylene oxide copolymers (PPO–PEO–PPO, also termed Poloxamers and Pluronics,) serve as thermosensitive hydrogels exhibiting well tolerability, low-level irritancy, and toxicity. Hence, they have been exploited in the biomedical arena, e.g., tissue engineering (Shim et al. 2005; Ju et al. 2013). At a specific composition, pluronics show dual behavior at lower or higher than its critical temperature. This critical temperature is introduced as lower critical gelation temperature (LCGT) where pluronics behave as a low viscous liquid, and at the higher temperatures of LCGT, one gradually encounters higher viscosity and the occurrence of gelification (Mayol et al. 2011). The appropriate and pertinent properties of Pluronics have led to their use in wound-healing applications (Khalil et al. 2007; Kant et al. 2014). To enhance the biocompatibility of platforms encompassing pluronics, they have been adorned with polysaccharides such as hyaluronic acid (HA) (Mayol et al. 2008, 2011). HA, a natural glycosaminoglycan, is the main component of the extracellular matrix (ECM) in mammalian connective tissues, vitreous, and nucleus pulposus wherein it fulfills both, the physiochemical and biological functions. Due to its important biological functions, it accelerates tissue regeneration, e.g., dermal skin. Skin repairs are promoted by HA and it has been employed in dermal engineering applications such as a dermal filler and wound dressings (Barbucci et al. 2002; Kablik et al. 2009). When these pluronic hydrogels are applied in combination with HA, the viscoelastic properties of the ensuing gel and its biocompatibility are improved. This condition occurs without affecting the gelling process, while improving the mucoadhesive properties which makes it easier to attach to the damaged tissues (Makvandi et al. 2019a, b).

However, a bacterial infection is a key and generally unsolved issue in regenerative medicine (Makvandi et al. 2020a). Thus, materials comprising antimicrobial compounds have been employed for infection therapy (Zare et al. 2020; Makvandi et al. 2020a; Jamaledin et al. 2020). Among assorted nanometals, AgNPs have demonstrated great potential for biomedical applications. For instance, AgNPs remarkably improve fibroblast proliferation, collagen synthesis, and cell adherence, showing their potential for wound healing (Li et al. 2017; Makvandi et al. 2020b; Wang et al. 2020). Among different approaches for the fabrication of silver NPs, the green nanotechnology strategy by deploying naturally occurring materials has garnered extensive attention in the medicinal sector as they avoid organic passivation that is perilous both for the environment and the human body (Makvandi et al. 2019a, b, 2021a).

Naturally occurring compounds present in extracts are potential candidates for the synthesis of AgNPs due to their minimal side effects (Ashrafizadeh and Ahmadi 2019; Mohammadinejad et al. 2019a; Sobhani et al. 2019), ease of availability, and large-scale synthesis (Mohammadinejad et al. 2019b). The plant extract-mediated synthesis of nanoparticles can protect the environment as the presence of biomolecules such as flavonoids, alkaloids, terpenoids, and sugars, etc. serve as reducing agents and capping agents rather than toxic hydrides or hydrazines (Makvandi et al. 2020a). Native plants such as ginger, cinnamon, mustard, and garlic illustrate different degrees of antimicrobial characteristics (Noman et al. 2016; Mahdy et al. 2017). Azadirachta indica (Neem) exhibits antibacterial and antifungal properties (Das 2014). Tea (*Camellia sinensis*) is widely used and cultivated as one of the most important industrial products in more than 45 countries with large consumption worldwide ~ 3 billion kilograms every year (Pang et al. 2016; Pastoriza et al. 2017). Tea, with its unique taste/flavor, has many health benefits due to its various bioactive components, including flavanols, polyphenols, catechins, amino acids, caffeine, vitamins, carbohydrates, and phenolic acids (Prasanth et al. 2019; Feng et al. 2020) with valuable biological and therapeutic effects such as antimicrobial, antioxidant, anti-inflammatory, neuroprotective, hepatoprotective, and cardioprotective (Zare-Zardini et al. 2020). Tea extract has been extensively explored for the preparation of wide-ranging nanoparticles of iron, silver, and copper among others (Nadagouda and Varma 2008; Hoag et al. 2009; Moulton et al. 2010; Nadagouda et al. 2010; Markova et al. 2014; Plachtová et al. 2018; Khatami et al. 2019).

The antibacterial activity of these nanoparticles has been exploited for usage in medical devices. To have access for maximum antibacterial effect, they have been integrated into multi-wall carbon nanotubes (MWCNTs) (Wang et al. 2020). Interestingly, MWCNTs are endowed with unique properties such as lightweight, chemical/thermal stability, antibacterial activity, highly tensile strength, excellent conductivity, and large surface area (Akbari et al. 2010; Murugesan et al. 2020). Pretreatment of MWCNTs generates oxygen-containing functional groups (such as OH and COOH) on its surfaces. Subsequently, the formation of nucleation sites acts as a home of Ag ions by ions-functional groups interactions and provides a growth situation for Ag NPs. Ag-decorated MWCNTs exhibited bacterial inactivation (Verma et al.; Seo et al. 2014). It has been illustrated that various molecules such as proteins, nucleic acids, and proteins can be loaded onto MWCNTs, with potential applications in biology and medicine, including the targeted delivery to cancer cells (Delfi et al. 2021; Makvandi et al. 2021a, b; Saeednia et al. 2019).

Herein, an easy, expeditious, and reproducible methodology is described for the greener synthesis AgNPs using *Camellia sinensis* extract and adorning the ensuing AgNPs on MWCNTs including their full characterization. Finally, the antimicrobial activity was evaluated against the Gram-positive (*S. aureus* and *E. faecalis*) and Gram-negative (*E. coli* and *Klebsiella*) bacteria with possible appliances in medicine. Afterward, we formulated the thermosensitive nanocomposite hydrogels based on Pluronics and HA and Ag NPs-decorated MWCNT for potential use as a wound care material.

## Experimental section

### Chemicals

The *Camellia sinensis* leaves were obtained from a local market (Ahvaz, Iran). Silver nitrate (99.98%) was purchased from Merck, Germany. MWCNTs, with 10 μm average length, were obtained from Sigma-Aldrich. To provide the highest dispersion, the MWCNTs suspension was stirred at 55 °C for approximately 7 h, and then, it was sonicated in an ultrasonic bath (50 Hz, 0.138 kW) at 70 °C for 10 min. All other materials were of commercial reagent grades.

### Preparation of green *Camellia sinensis* leaves extract

**Fig. 1:**
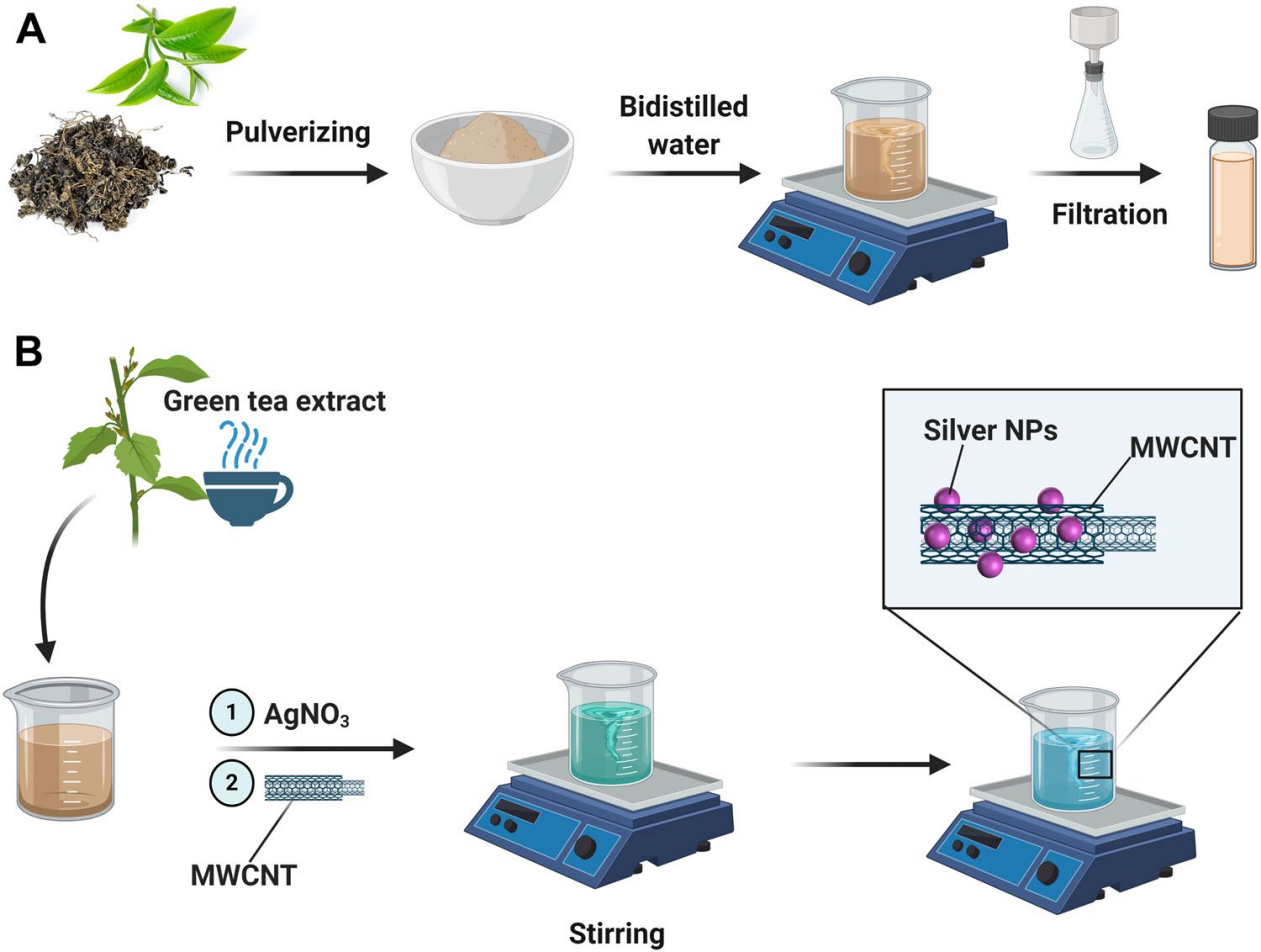
**a** Schematic presentation for the preparation of tea extract; **b** illustration for the fabrication of AgNPs-decorated MWCNT nanocomposites

*Camellia sinensis* leaves of 50 g were washed with deionized water to eliminate the dust and dried in an oven at 40 °C. The dried leaves were ground into a fine powder (mesh size 60 μm), pulverized in a knife mill, and sieved into a particle size of 0.4 μm and, then, kept refrigerated in glass containers before further processing. Next, the powder was added to 500 mL of deionized water and the solution was stirred and heated to 80 °C for 20 min. Then, the mixture was filtered through Whatman No. 42 filter paper to remove any residual powder. Finally, the extract was stored at 4 °C until it was used for the experiments (Onitsuka et al. 2019). Schematic illustration for the tea extraction process is depicted in Fig. 1a.

### Synthesis of silver nanoparticle

The biosynthesis of AgNPs was conducted by employing a greener approach in an organic solvent-free medium. Briefly, 50 mg of dried *Camellia sinensis* leaves extract was mixed with 47 mL water, and its pH was adjusted to 9 using NaOH. Then, under constant stirring of the solution (1000 rpm), silver nitrate aqueous solution (3.4 μg/mL) was gradually added and the contents stirred at room temperature (25 °C) for 20 min. The biosynthesized AgNPs in the extract was stored at 4 °C for further characterization (Khorrami et al. 2018).

### Synthesis of silver nanoparticle-decorated MWCNTs

MWCNTs were first treated with a mixture of acids with concentrated H_2_SO_4_ and HNO_3_ (3:1, v/v) at 60 °C for 3 h. For the synthesis of Ag-MWCNTs, 20 mg of MWCNTs was dispersed in 50 mL of H_2_O and sonicated for 30 min. It was then mixed with 50 mL AgNO_3_ (3.4 μg/mL) and sonication was continued for 20 min. Afterward, 50 mL of *Camellia sinensis* leave extract, as a reducing and stabilizing agent, was gradually added to the mixture under vigorous stirring. Finally, the biosynthesized nanoparticle-decorated MWCNTs were stored at 4 °C for further characterization. The schematic of the fabrication of the process is shown in Fig. 1b.

### Measurements

To evaluate and monitor the biosynthesis of AgNPs, a UV–Vis spectrophotometer (Cary 50, Australia) was used and was performed at a resolution of 1 nm and wavelength range between 300 and 600 nm. The morphologies of the AgNPs and AgNPs-decorated MWCNTs were ascertained by TEM (Jeol JEM-ARM200CFEG). The TEM samples were prepared by sonicating each sample for 15 min and placing a drop onto carbon-coated copper grids. Then, each grid was dried in the laboratory air at room temperature.

### HPLC analysis

The *Camellia sinensis* leaves’ extract (200 mg) was added to 20 mL methanol solution 80% and mixed overnight in the dark. Then, the mixture was centrifuged at 3000 rpm for 10 min. The supernatant solution was decanted and stored for performing the HPLC analysis. The analysis was recorded using an HPLC system (Agilent 1200 series model, San Jose, CA, USA) equipped with a diode array detector for monitoring all wavelengths (227–550 nm) and C18 column (250 mm × 4.6 mm i.d., 5 µm, Phenomenex, USA) at 40 °C. Chemical Station software (Rev B. 03. 02, Agilent Technologies) was also applied for the data acquisition; injection volume being 20 µL. The mobile phase comprised orthophosphoric (1% w/v, A) and acetonitrile (≥ 99.9%, B) in the following gradient elution program (flow rate: 1 ml min^−1^). 10–57 min, B (8%) increase to 18%; at 78 min, B (24%); at 80 min, B (26%); at 92 min, B (28%); at 92 min, B (28%); at 98 min, B (80%) and finally at 108 min, B (8%). The concentration of standard solutions encompassed 0.1 mg mL ^−1^ [for (-)-epigallocatechin (EGC)], 0.01 mg mL ^−1^ [for (-)-epicatechin (EC)], and (-)-catechin gallate (CG) in 80% aqueous methanol. Moreover, (-)-gallocatechin (GC), (-)-gallocatechin gallate (GCG), (-)-epicatechin gallate (ECG), and (-)-epigallocatechin gallate (EGCG) standard solutions were prepared with 80% aqueous methanol to meet the concentration of 0.05, 0.025, 0.05, and 0.5 mg mL ^−1^, respectively. The theobromine, caffeine, and rutin solutions were also 0.01, 0.1, and 0.01 mg mL ^−1^.

### Antibacterial evaluation

The in vitro antibacterial activity of the samples was assayed using a direct contact test with agar diffusion. Gram-positive (*S. aureus* and *E. faecalis*) and Gram-negative (*E. coli* and *Klebsiella*) bacteria were used to assess the antibacterial activity of the prepared nanoparticles. Standard bacterial strains were purchased from Pasteur Institute, Iran. To examine the antibacterial activity of Ag and Ag/MWCNT, we used the primary concentration of each sample. For Gram-negative bacteria (*E. coli* and *Klebsiella*), tetracycline was used. For negative control, we used a disc containing sterile distilled water. A positive control is a solution of ampicillin agar plate. Antibacterial activity was evaluated by measuring the diameter of the inhibition zone (mm) on with a concentration of 100 mg mL^−1^. All tested bacteria were maintained in Muller–Hinton broth (Merck). The agar plates were inoculated from the standardized cultures of the test organisms using a sterile cotton swab to then spread as uniformly as possible throughout the entire media. The tablet (diameter: 9 mm) was introduced on the upper layer of the seeded the surface of plates and the results were reported as mean ± standard deviation (SD) after three repeats.

### Preparation of thermosensitive hydrogel

The hydrogels were prepared by adding Pluronics F127 (15% wt) and F68 (15% wt) to the aqueous dispersion of Ag NPs/MWCNT by mixing under continuous stirring at 4 °C. Subsequently, HA was added to this mixture at room temperature to obtain the concentration of 1% wt of HA. The composition of the hydrogels was optimized by rheological analysis to obtain a gelification temperature (T_gel_) around body temperature (Makvandi et al. 2019a, b).

### Rheological properties

Small-amplitude oscillatory shear tests were performed to evaluate the time-dependent response of the thermosensitive hydrogels and their linear viscoelastic properties, i.e., G″ and G′; frequency was in the range from 0.01 to 10 Hz. The measurements were carried out through a rotational rheometer (Mars III, HAAKE Rheometer, Waltham, MA, USA), using a parallel plate geometry. The tests were performed at the controlled temperatures of 20 and 40 °C using a thermostatic bath. To identify the linear viscoelastic response range of the materials, preliminary strain sweep tests were performed on the samples, at the oscillation frequency of 1 Hz. The tests were repeated at least three times for each sample. The gelation temperature of the formulation was evaluated by monitoring the viscoelastic parameters (G′ and G″) as a function of the temperature ranging from 25 to 40 °C at a fixed oscillation frequency of 0.01 Hz. During all the tests, the samples were placed into a chamber properly designed to avoid the evaporation of the solvent. For the viscosity analysis, a steady-state shear test in terms of flow curves was performed to evaluate the dependence of viscosity upon the shear rate (Makvandi et al. 2019a, b).

## Results

### Synthesis and characterization

**Fig. 2:**
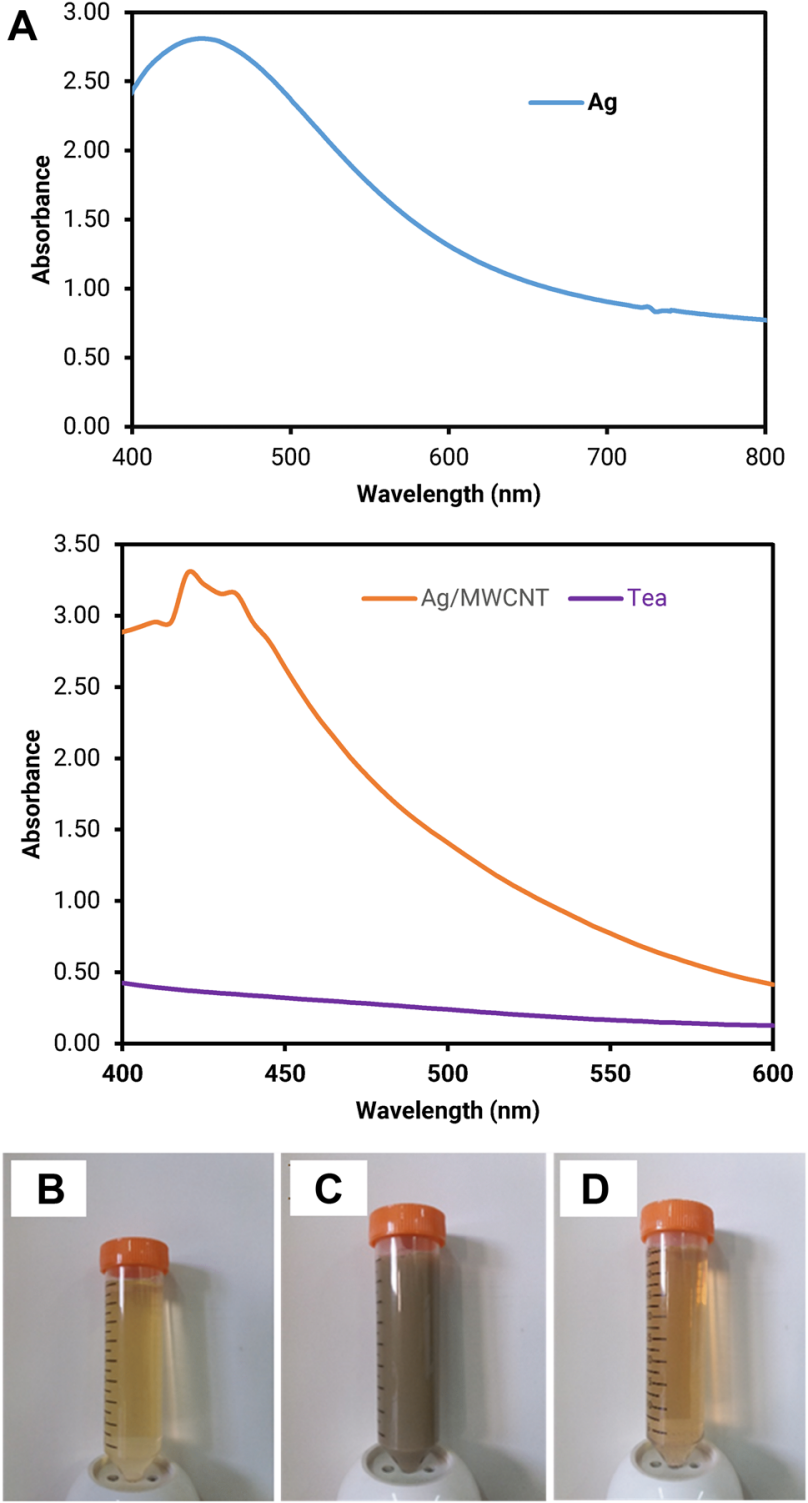
**a** UV–Vis spectra of AgNPs, tea extract, and AgNPs decorated on MWCNTs; the color of **b** tea extract **c** biosynthesized AgNPs and **d** biosynthesized AgNPs decorated on MWCNTs

To determine whether AgNPs were biosynthesized by *Camellia sinensis* extract, the UV–Vis spectra were recorded after each part of the synthesis process. As can be seen from Fig. 2a, the maximum absorbance for AgNPs appeared at a wavelength of 480 nm, indicating the formation of AgNPs (Makvandi et al. 2019a, b). After 3 h of initiation of the reaction, the reduction of silver ions to nanoparticles was completed in approximately 24 h. After exposure to *Camellia sinensis*, the reduction of Ag^+^ to Ag^0^ occurred, resulting in a color alteration of solution from colorless to yellow; this color alteration is a result of the excitation of surface plasmon vibration with the AgNPs. Figure 2b–d displays the color alteration of silver nitrate to AgNPs in the absence and presence of MWCNTs.

**Fig. 3:**
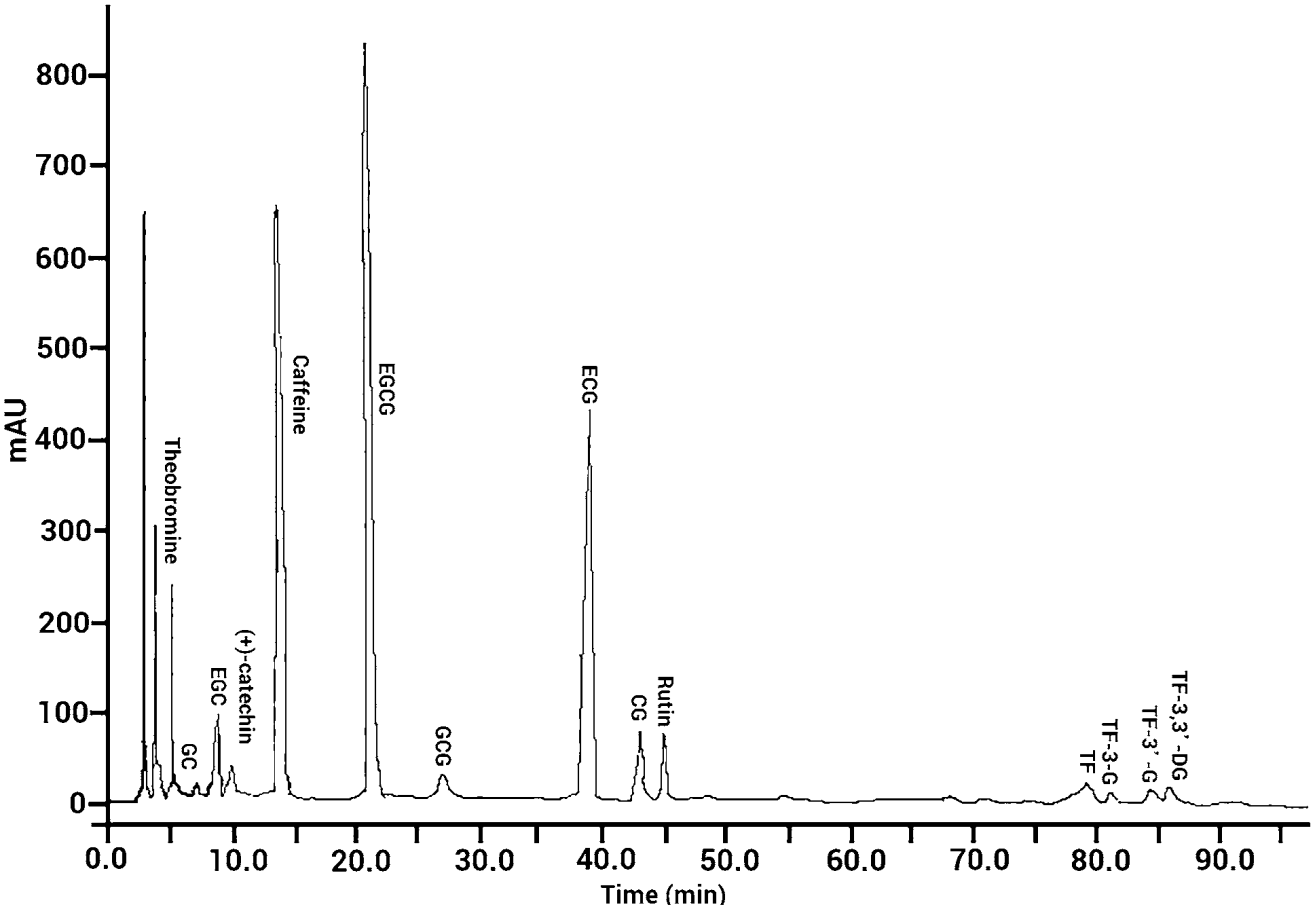
HPLC chromatogram of extractable ingredients from green tea

HPLC methodology has been conducted for the simultaneous analysis of *Camellia sinensis* constituents such as polyphenols, flavonoids, purine alkaloids, etc. (Nishitani and Sagesaka 2004; Sharma et al. 2005). The reproducibility of the green synthesis method was evaluated through the investigation of the absorption spectra of AgNPs. The localized surface plasmon resonance (LSPR) of the synthesized NPs was recorded for five tea extracts; absorption spectra compared with each other revealed no significant difference in LSPR. This confirms that the tea extract (as a bio-reducing agent) possesses enough bioactive components (viz., flavonols, polyphenols, catechins, amino acids, caffeine, vitamins, carbohydrates, and phenolic acids) in each of the examined *Camellia sinensis* leaves’ extracts. The extractable components in green tea through HPLC analysis are shown in Fig. 3 with the total alkaloid, polyphenol, and theaflavins of green tea being listed in Table 1. The major alkaloid was found to be caffeine. The catechin compounds (viz., EGCG, ECG, EGC, GC, and CG) were identified and the highest concentration belonged to EGCG.

**Table 1:** The alkaloid, polyphenol, and theaflavin components of green tea (mg g^−1^)

Compounds	Mean ± SD
Total alkaloids (theobromine, caffeine)	32.06 ± 0.271
Total catechins (GC, EGC, EC,EGCG,GCG,CG)	153.8 ± 0.271
Total theaflavins (TF, TF-3-G, TF-3′-G, TF-3,3′-DG)	1.72 ± 0.061

**Fig. 4:**
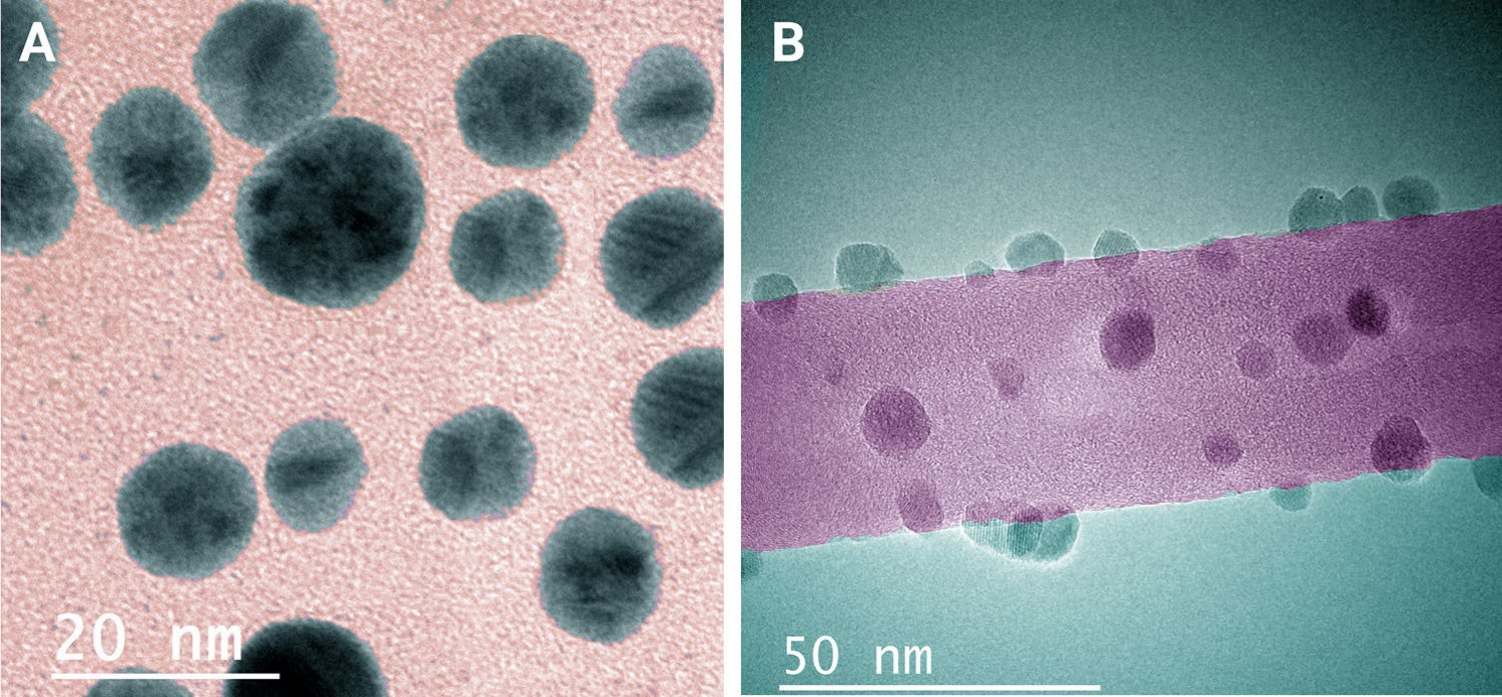
TEM images of **a** biosynthesized AgNPs and **b** MWCNTs decorated with biosynthesized AgNPs

**Fig. 5:**
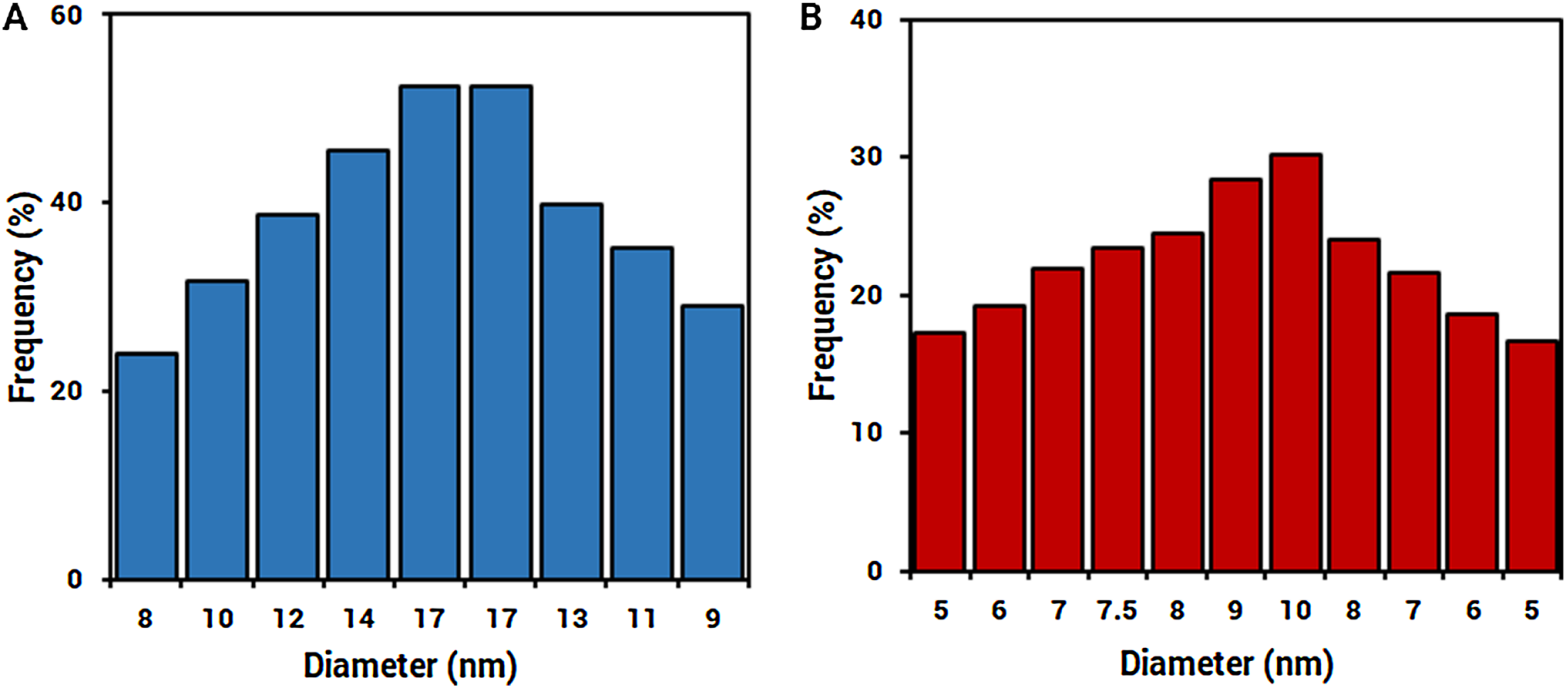
The size distribution graph of AgNPs (**a**) and AgNPs decorated on MWCNTs (**b**)

Oxygenated functional groups on the MWCNTs serve as the active sites for loading Ag ions, their nucleations, and uniform distribution (Ebbesen 1996); AgNPs decorated on the MWCNTs were dispersed all over the MWCNTs surfaces, and their morphology size and shape were assessed by transmission electron microscopy (Mohseni-Dargah et al. 2019). Figure 4 exhibits the TEM image of the prepared AgNPs (at the scale of 50 nm) and AgNPs decorated on MWCNTs (at the scale of 20 nm). Also, the average size distribution graphs of AgNPs (A) and AgNPs decorated on MWCNTs were determined by Digimizer software as depicted in Fig. 5.

### Antibacterial activity

**Fig. 6:**
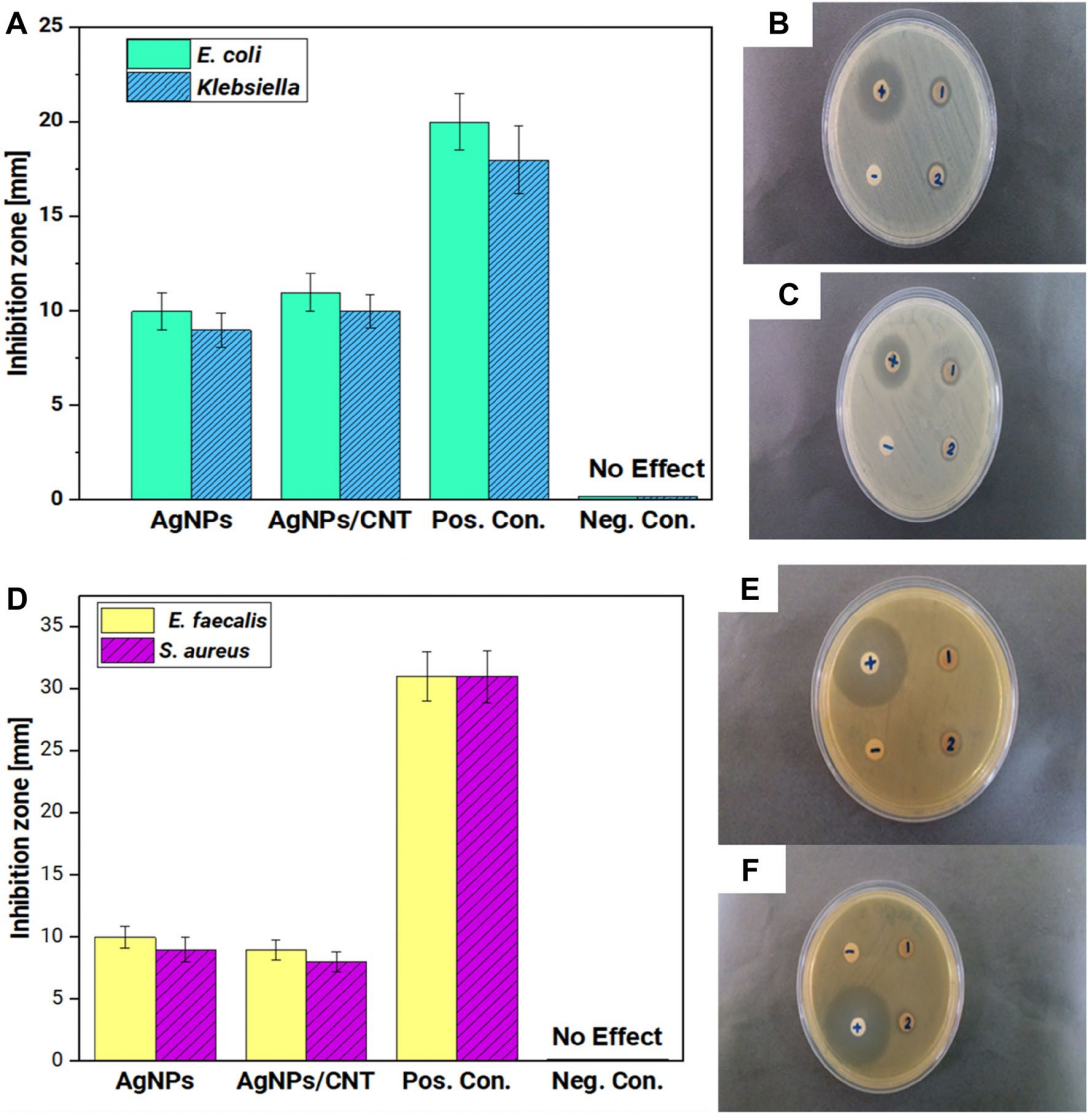
Qualitative antibiogram test of biosynthesized NPs for **a** Gram-negative bacteria and the antibacterial property of multi-walled carbon nanotubes decorated with biosynthesized AgNPs on **b** *Escherichia coli*, **c** *Klebsiella*; qualitative antibiogram test of biosynthesized NPs for **d** Gram-positive bacteria and antibacterial property of multi-walled carbon nanotubes decorated with biosynthesized AgNPs on **e** *Enterococcus faecalis* and **f** *Staphylococcus aureus*

Figures 6 demonstrates the antibacterial activity of green-synthesized nanoparticles after exposing Gram-negative and Gram-positive bacteria. All samples showed antibacterial effect toward Gram-negative bacteria. As can be seen, the antibacterial activity of both AgNPs and Ag/MWCNT is higher for Gram-negative bacteria, compared to the Gram-positive bacteria. Besides, the antibacterial activity of Ag/MWCNT is slightly higher than Ag alone toward Gram-negative bacteria (Fig. 6). As expected, ampicillin showed a higher inhibition zone than the biosynthesized nanomaterials and negative controls did not show any antibacterial activity.

### Rheological behavior

**Fig. 7:**
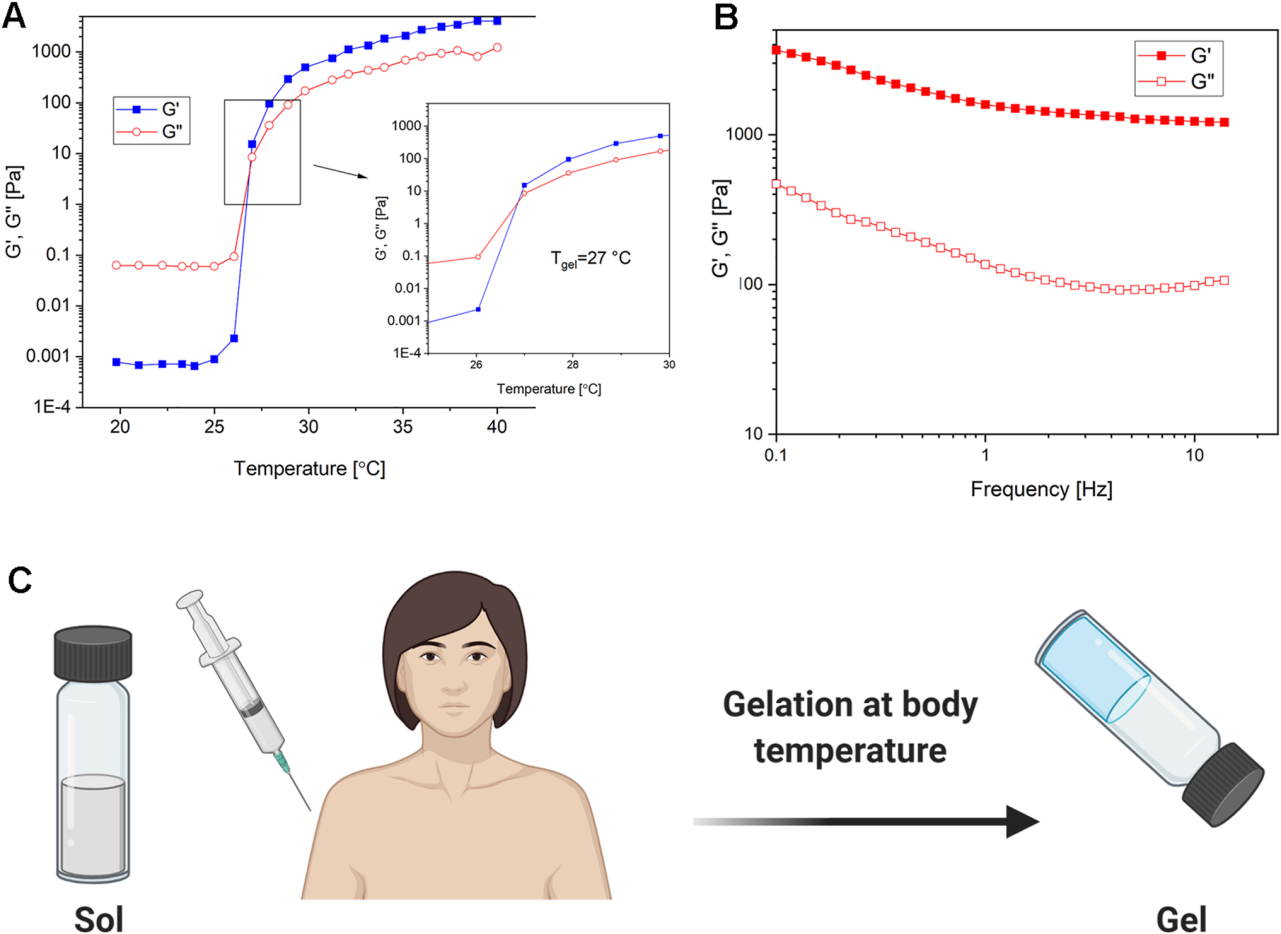
**a** The sol–gel phase transition by rheological experiments. **b** Mechanical spectra of the hydrogels after gelation (40 °C).** c** Schematic illustration of the injectable platform and gelling at body temperature

To perceive the gelation behavior of solutions, rheological measurements were undertaken. The transformation temperature of hydrogels from viscous (G″ > G′) to elastic (G′ > G″) behavior is named a gelation temperature (T_gel_). The viscoelastic properties of hydrogels alter by a gradual increase in the temperature and thus provide an ability to evaluate the behavior of the gel. The temperature range of 20–40 °C was selected for the viscoelastic investigation. The gel point was obtained from the intersection of two elastic (G′) and viscous moduli (G″) (Mayol et al. 2008). Both moduli (elastic and viscous) of test samples at a specific frequency (0.1 Hz) are shown against the temperature in Fig. 7a**.** The examination of different concentrations (10–30% wt) of Pluronic aqueous solutions, viz., F127 or F68 exhibited no gelification process at a T_gel_ close to the body temperature (T_b_). In contrast, their mixture (F127/F68) with specific concentration ratios generates a hydrogel blend that possesses a gelling temperature close to the T_b_. The mechanical spectra, G’ and G’’ as a function of frequency at a higher temperature than T_gel_, are shown in Fig. 7b. According to the figure, the elastic modulus is higher than the viscous modulus at 40 °C and shows the rheological behavior of a gel-like material, because the performance of both moduli at this temperature is independent of the frequency.

**Fig. 8:**
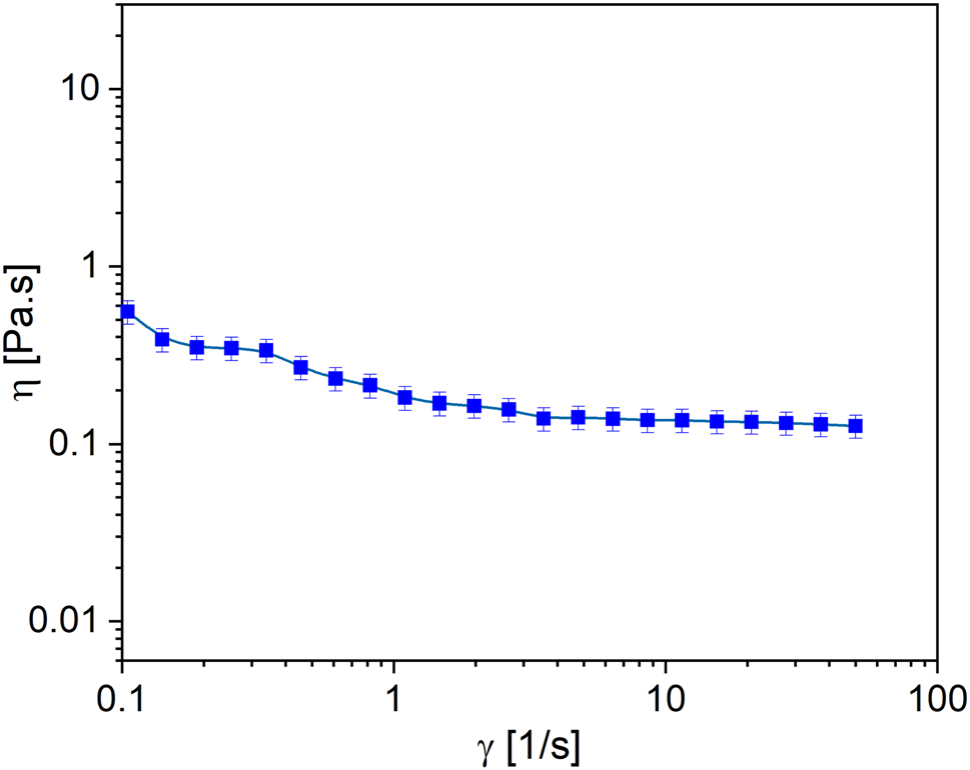
The viscosity effect of the thermosensitive hydrogel against the various shearing rate at room temperature

The shear-thinning and viscosity behavior of prepared hydrogel at 25 °C is shown in Fig. 8. The viscosity decreases with increasing shear rate until it reaches a constant pseudo-Newtonian behavior at high shear rates. Polymeric solutions and physically bonded gels behave in such a way, which can be due to the breaking of physical bonds and topological interactions between the polymer chains under shearing. The fracture of the bonds increases the chain mobility and reduces the abrasion between the polymer and solvent moieties within the hydrogel bed. For easier injection of materials through the needle, it is better to have a higher shearing rate and lower viscosity (Mayol et al. 2011; Dessi et al. 2013).

## Discussion

The synthesis of nanoparticles employing chemicals has been known for a while but invariably deploys toxic substances and organic solvents that are detrimental to the environment and humans (Pattabi and Uchil 2000). In contrast, the biosynthesis of NPs has been considered a growing need for the development of eco-friendly technologies (Varma 2012; Hebbalalu et al. 2013), the use of safer and renewable materials instead of toxic chemicals (Patra and Baek 2014; Zare et al. 2019).

Herein, the AgNPs are generated by safer entities present in tea extract which are concurrently formed on the MWCNTs by reduction of Ag ions; prior to that, acid treatment is employed for forming functional groups on the surface of MWCNTs. for synthesis of Ag-decorated MWCNTs. The functional groups (such as COOH) on the MWCNTs improve the stability and chemical reactivity (Hamouda et al. 2021). Hence, the precise and controlled nuclei formation of Ag NPs on the surface of MWCNTs, strong Ag-carbon adhesion, and high dispersion forces between MWCNTs and Ag NPs can be achieved.

The reproducibility of our green protocol was also evaluated by a three-time analysis of extract using HPLC instrument which resulted in the reproducible data. The spherical shapes of these NPs were confirmed by TEM, while the MWCNTs improved the dispersibility of NPs and assisted in generating smaller NPs.

Antibacterial activity of nanoparticles has led to their application in the field of medicine and prevention of bacterial resistance and our AgNPs adorned MWCNT displayed a higher antibacterial effect against Gram-negative bacteria (Fig. 6). It has been demonstrated that multifunctional nanoparticles containing caffeic acid, phenethyl ester, and juglone have high antibacterial activity against *S. aureus* and *E. coli* (Durak et al. 2020). It is worth mentioning that the wall of bacteria inhibits the entrance of antibacterial agents and stimulation of their effect. Green synthesized AgNPs using *Artocarpus hirsutus* extract showed high antibacterial activity against Enterobacter aerogenes and listeria monocytogenes which emanates from the ability of nanoparticles to penetrate the bacterial cell wall (Dhand et al. 2016). Despite the high antibacterial activity, the nanoparticles are biocompatible and display no or low toxicity against normal cells (Guo et al. 2020) as exemplified by plant-derived chemicals such as curcumin into silica nanoparticles with enhanced antibacterial activity (Mirzahosseinipour et al. 2020). Overall, these studies highlight the fact that nanostructures are capable of reducing and inhibiting the growth of bacteria and can be applied instead of conventional antibacterial agents to suppress bacterial resistance (Li et al. 2020; Zhen et al. 2020).

**Fig. 9:**
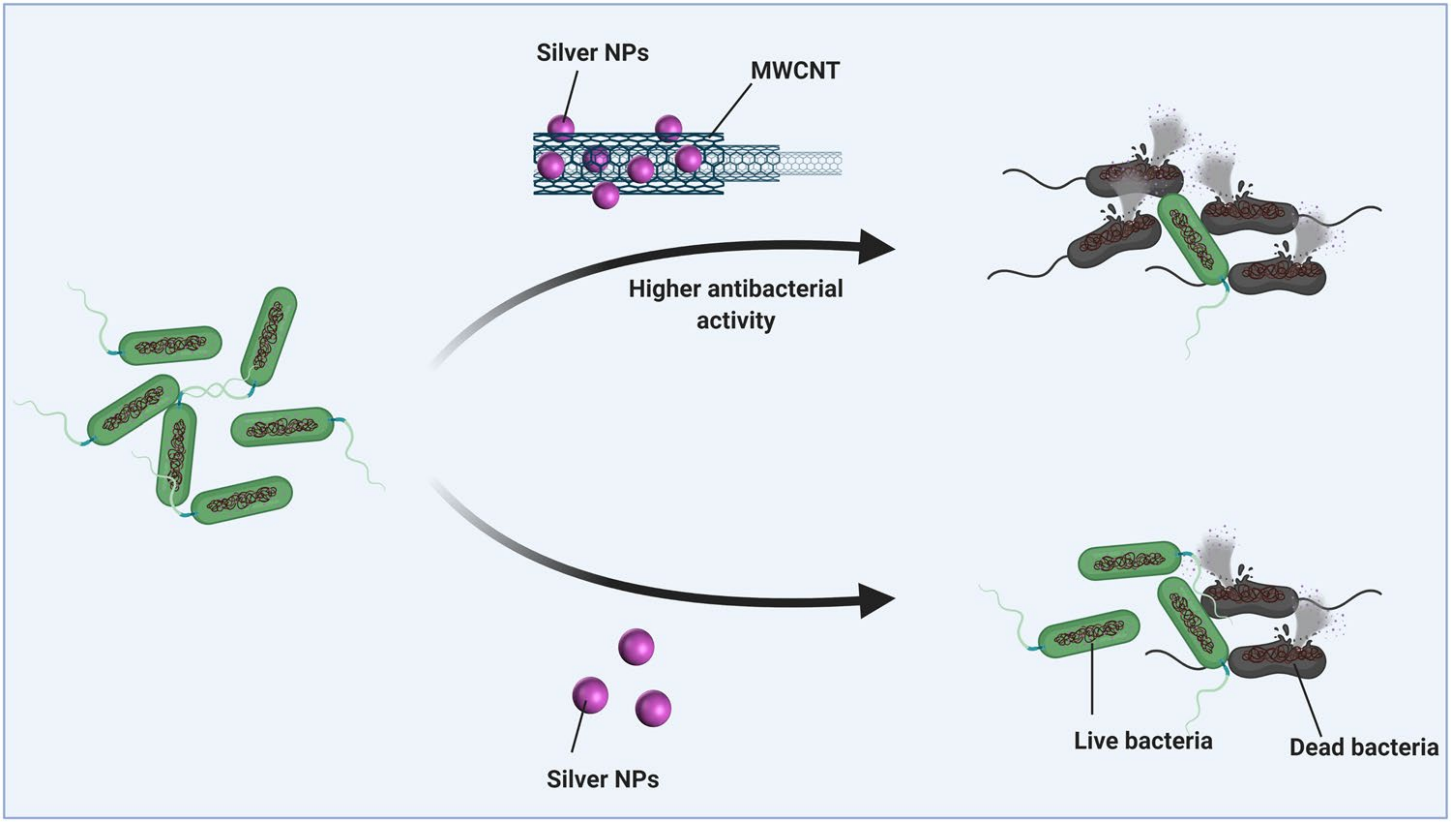
Schematic illustration of antibacterial activity of biosynthesized AgNPs and Ag/MWCNT against Gram-negative bacteria

The antibacterial activity of AgNPs has been applied in different fields of medicine. For instance, after bone graft introduction in alveolar ridge construction, there is a risk of infection which may inhibit the formation of bone; silver nanoparticle-coated collagen membrane can stimulate antibacterial activity (Chen et al. 2018). It has been demonstrated that *S. aureus* and *Pseudomonas aeruginosa* are factors that negatively affect bone substitution and after using AgNPs, the activity and growth of these bacteria undergo inhibitory effect, while these nanocarriers being biocompatible exert no adverse effects on normal cells. Using antibacterial drugs would result in the development of resistance and it is necessary to identify novel methods for combating such bacteria (Makvandi et al. 2021b). AgNPs are promising agents in overcoming the bacterial infection with minimal chance of bacterial resistance due to minuscule concentration of AgNPs. Ambi and colleagues synthesized AgNPs with antibacterial activity (Ambi et al. 2018). Bacterial colonization stimulates the activation of these AgNPs and it was found that they can effectively suppress the growth of Gram-positive and Gram-negative bacteria such as *Staphylococcus epidermis*, methicillin-resistant *Staphylococcus aureus,* and *E. coli*. Shao and colleagues prepared AgNP-decorated graphene nanocomposite and assessed its antibacterial activity (Shao et al. 2015); they exerted antibacterial impact against Gram-negative *E. coli* ATCC25922 and Gram-positive *S. aureus* ATCC6538 bacteria. Notably, it appears that the addition of multi-walled carbon nanotubes is an efficient strategy in improving the antibacterial activity of AgNPs. In the present study, AgNPs decorated MWCNTs display higher antibacterial activity relative to AgNPs; antibacterial activity of biosynthesized AgNPs and Ag/MWCNTs against Gram-negative bacteria is schematically depicted in Fig. 9.

In this research, temperature-sensitive hydrogels were prepared wherein AgNPs decorated on MWCNTs using *Camellia sinensis* extract. The gel behavior of blending hydrogel occurs near the body temperature which is a significant advantage. The gelation process of Pluronic transpires without any need for toxic crosslinker or organic solvent, and hence, it can be applied in a biological application; the use of HA, the second component of a hydrogel, magnifies the biocompatibility of hydrogel. The polymer interactions between HA and pluronic followed by micelle generation and self-assembling process clarify the path of the possible underlying mechanism (Mayol et al. 2011).

## Conclusion

In the present study, we have successfully synthesized AgNPs adorned on MWCNTs using *Camellia sinensis*. Notably, the method of preparation of nanoparticles was eco-friendly and synthesized nanoparticles demonstrated superb properties in terms of their spherical shape with maximum absorbance at 480 nm and antibacterial activities. *Camellia sinensis* leaf extract was used as a reducing agent for the in situ synthesis of AgNPs due to its valuable biological and therapeutic effects such as antioxidant, anti-inflammatory, anti-tumor, hepatoprotective, and cardioprotective properties with barely any side effects. Finally, the antibacterial activity of nanoparticles against Gram-positive (*S. aureus* and *E. faecalis*) and Gram-negative (*E. coli and Klebsiella*) bacteria was investigated, and it was found that these nanocarriers have a great antibacterial activity and can effectively inhibit the growth of bacteria. We have also introduced a thermosensitive and injectable hydrogel for potential medical applications in the biomedical sector. The rheological behavior of the prepared hydrogel was examined to investigate its viscoelastic moduli.
